# Controlling Your “App”etite: How Diet and Nutrition-Related Mobile Apps Lead to Behavior Change

**DOI:** 10.2196/mhealth.7410

**Published:** 2017-07-10

**Authors:** Joshua H West, Lindsay M Belvedere, Rebecca Andreasen, Christine Frandsen, P Cougar Hall, Benjamin T Crookston

**Affiliations:** ^1^ Department of Health Science Brigham Young University Provo, UT United States

**Keywords:** diet, nutritional status, mobile apps, behavior and behavior mechanisms

## Abstract

**Background:**

In recent years, obesity has become a serious public health crisis in the United States. Although the problem of obesity is being addressed through a variety of strategies, the use of mobile apps is a relatively new development that could prove useful in helping people to develop healthy dietary habits. Though such apps might lead to health behavior change, especially when relevant behavior change theory constructs are integrated into them, the mechanisms by which these apps facilitate behavior change are largely unknown.

**Objective:**

The purpose of this study was to identify which behavior change mechanisms are associated with the use of diet- and nutrition-related health apps and whether the use of diet- and nutrition-related apps is associated with health behavior change.

**Methods:**

A cross-sectional survey was administered to a total of 217 participants. Participants responded to questions on demographics, use of diet and nutrition apps in the past 6 months, engagement and likability of apps, and changes in the participant’s dietary behaviors. Regression analysis was used to identify factors associated with reported changes in theory and separately for reported changes in actual behavior, after controlling for potential confounding variables.

**Results:**

The majority of study participants agreed or strongly agreed with statements regarding app use increasing their motivation to eat a healthy diet, improving their self-efficacy, and increasing their desire to set and achieve health diet goals. Additionally, majority of participants strongly agreed that using diet/nutrition apps led to changes in their behavior, namely increases in actual goal setting to eat a healthy diet (58.5%, 127/217), increases in their frequency of eating healthy foods (57.6%, 125/217), and increases in their consistency of eating healthy foods (54.4%, 118/217). Participants also responded favorably to questions related to engagement and likability of diet/nutrition apps. A number of predictors were also positively associated with diet-related behavior change. Theory (*P*<.001), app engagement (*P*<.001), app use (*P*<.003), and education (*P*<.010) were all positively associated with behavior change.

**Conclusions:**

Study findings indicate that the use of diet/nutrition apps is associated with diet-related behavior change. Hence, diet- and nutrition-related apps that focus on improving motivation, desire, self-efficacy, attitudes, knowledge, and goal setting may be particularly useful. As the number of diet- and nutrition-related apps continues to grow, developers should consider integrating appropriate theoretical constructs for health behavior change into the newly developed mobile apps.

## Introduction

Currently, 68% of men and 64% of women in the United States are considered overweight or obese [1,2]. Much of this increase can be linked to cheap and unhealthy food production coupled with increased consumption of foods with minimal nutritional value [3]. In addition to obesity, these changes in dietary patterns are associated with many other serious health conditions, including hypertension, stroke, heart disease, elevated cholesterol, and diabetes [4]. Efforts to address the complex dilemma of obesity in the United States typically include the promotion of physical activity and healthy nutritional and dietary habits [5].

With the advent of mobile phone technology, a vast number of health-related mobile apps have been developed and are now being widely used to tackle health problems [6]. Many such apps provide users additional methods for monitoring their health or achieving health-related goals. The proliferation of health-related apps represents a potential resource in addressing obesity [7]. Studies have shown that using health-related apps can successfully lead to health behavior change related to weight loss or weight management [8,9]. Many health care providers see the potential of health-related apps to facilitate weight management and encourage patients to use them [6,10].

An assortment of apps has been developed to help individuals monitor their food consumption through calorie counting or food diary approaches [6,8]. Other apps provide individuals with healthy diet and nutrition facts and information (eg, MyFitnessPal, FitBit, and Lose It!) [8,11]. Studies have found that the use of diet-related apps can lead to improved diet and nutrition health behavior change [9]. Turner-McGrievey et al [9] found that participants using diet apps consumed fewer calories than those using a website or paper journal method for monitoring dietary intake. Similarly, when comparing the use of diet-related apps with website groups and food intake diary groups in a randomized control trial, participants using the apps had higher retention, adherence, and weight loss [12].

Previous research has found that interventions integrating models of behavior change theory may be effective. Inclusion of constructs from established health behavior change theories increases the effectiveness of planning, implementing, and evaluating interventions [13]. Three leading health behavior change theories for nutrition education and intervention are the health belief model (HBM), theory of planned behavior (TPB), and social cognitive theory (SCT) [14]. The HBM has been widely used since the 1950s and includes primary constructs that predict “if” and “why” a person will take action toward detecting, preventing, and controlling unhealthy behaviors. This is achieved through perceived susceptibility and severity, as well as the barriers and benefits associated with engaging in certain behaviors [13]. Constructs that examine individual motivation and attitudes and how they determine the likelihood of performing specific behaviors are the focus of TPB [13]. The SCT identifies the ever-present tension between human agency and social structure [13]. A key advantage of SCT is how it accounts for both individual decision making and environment through the concept of reciprocal determinism, which in turn leads to a deeper sense of self-efficacy through personal experiences, persuasion, and vicarious learning [13]. Provided the complexities associated with nutrition behaviors, a practical approach to behavior change may involve a combination of distinct constructs and elements from each theory effectively forming a polytheoretical approach [14].

Studies have shown that using health apps for diet can successfully lead to positive changes in weight management [12]. Health apps have the potential to decrease some barriers to traditional prescriptions for behavior change, including expense, patient burden, and variable adherence. Specifically, how engaging, convenient, and easy to use the app is can be a mechanism for reducing barriers and increasing adherence. However, none of the leading weight loss apps have been evaluated in a clinical trial, which underscores the need for a descriptive study such as this one to establish associations warranting further investigation [15,16]. Though several theories related to health behavior change are well established and generally accepted, recent content analyses of diet-related apps demonstrate that many diet-related apps have insufficient evidence-informed content or are lacking in theoretical constructs considered important in facilitating behavior change [17-19]. To date, no research has explored the specific mechanisms by which diet-related apps actually generate changes in behavior. The purpose of this study was to explore which behavior change mechanisms are associated with use of diet- and nutrition-related health apps and to examine whether the use of these apps is associated with actual changes in dietary behaviors.

## Methods

### Design and Procedure

This study consisted of a cross-sectional survey to assess the use of diet and nutrition apps in the past 6 months. A Qualtrics survey was distributed via Amazon Mechanical Turk (MTurk) to 239 participants. The survey was open on MTurk twice, once for approximately 2 weeks with a US $1 incentive (89 respondents) and then a second time for a few more weeks with a US $2 incentive (150 respondents). An advantage of Web-based data collection is the ability to access a wide variety of participants that represent a diverse sampling of those using the method of Internet communication [20]. It is projected that data collection through electronic sources will continue to rise because of potential outreach [21]. A typical MTurk sample is representative of the population criterion of interest for this research project.

### Sample

A number of inclusion and exclusion criteria were used to define the survey sample. Survey participants had to be at least 18 years of age, live in the United States, and be able to read English. Participants were excluded if they had not used a diet or nutrition app in the last 6 months or if they failed to complete all 16 survey questions. A total of 239 participants responded, of which 217 individuals met all requirements and completed all questions.

### Measurement

Participants responded to questions on demographics (eg, age, race, education and income level, state of residence), use of diet/nutrition apps in the past 6 months, engagement and likability of the apps, and changes in the participant’s dietary behaviors. A 5-point Likert-type scale was used to generate response categories for the variables related to behavior change.

The study used three health behavior theories to formulate the Likert-type scale survey questions focusing on mechanisms of behavior change and actual diet/nutrition-related behaviors. Questions based on SCT included those that measured outcome expectations, self-efficacy, subjective norms and knowledge, whereas questions based on TPB measured behavioral beliefs, intentions, attitudes, desires, normative beliefs, and goal setting. Finally, one question based on HBM measured perceived benefits. A composite total theory variable was constructed to provide a global estimate of changes in theory-related constructs. A polytheoretical measure was determined to be in line with the viewpoint that behaviors relating to diet/nutrition are too complex for any one single theory [14]. The Cronbach alpha coefficient for this composite variable was .941 (behavior=.842; engagement=.875). This variable was not normally distributed. Hence, a square root transformation was used.

### Statistical Analysis

Stata version 14 (StataCorp) was used to calculate all statistics. Descriptive statistics were calculated for each of the demographic, theory, engagement, and behavior variables. Multiple regression analysis was used to identify factors associated with reported changes in theory and separately for reported changes in actual behavior, after controlling for potential confounding variables.

## Results

The majority of study participants were white (83.9%, 182/217) and between the ages of 26 and 34 (44.2%, 96/217; Table 1). Most participants (87.5%, 190/217) had at least some college education. Just over half of the participants (55.8%, 121/217) were female, and 41.5% (90/217) were from the Southern United States.

Most (59.9%, 130/217) strongly agreed with the statement that using diet/nutrition apps increased their motivation to eat a healthy diet, whereas an additional 36.8% (80/217) agreed with the same statement (Table 2). Many respondents indicated that the apps improved self-efficacy: 43.8% (95/217) strongly agreed and 42.4% (92/217) agreed with the statement that using diet/nutrition apps increased their ability to eat a healthy diet, and 43.3% (94/217) strongly agreed and 48.4% (105/217) agreed with the statement that using diet/nutrition apps increased their confidence that they can eat a healthy diet. More than half (59.0%, 128/217) of the participants strongly agreed that using diet/nutrition apps increased their desire to set goals to eat a healthy diet, whereas 51.1% (111/217) strongly agreed that using diet/nutrition apps increased their ability to achieve their healthy diet goals.

**Table 1:** Summary of participant demographics.

Demographics		Frequency, n (%) (N=217)
**Age in years**		
	18-25	16 (7.4)
	26-34	96 (44.2)
	35-54	90 (41.5)
	55-64	11 (5.1)
	65 or over	4 (1.8)
**Race**		
	American Indian	2 (0.9)
	Asian	15 (6.9)
	Black/African American	17 (7.8)
	Native Hawaiian/Other Pacific Islander	1 (0.5)
	White	182 (83.9)
**Ethnicity**		
	Hispanic/Latino	13 (6.0)
	Non-Hispanic/Non-Latino	204 (94.0)
**Gender**		
	Male	96 (44.2)
	Female	121 (55.8)
**Education level**		
	Less than high school	1 (0.5)
	Diploma/GED	26 (12.0)
	Some college	56 (25.8)
	2-year degree	23 (10.6)
	4-year degree	91 (41.9)
	Master’s degree	18 (8.3)
	Professional degree (MD, JD)	2 (0.9)
**Region of residence in USA**		
	West	49 (22.6)
	South	90 (41.5)
	Midwest	36 (16.6)
	Northeast	42 (19.4)
**Household income, in US dollars (2016)**		
	Less than 30,000	50 (23.0)
	30,000-39,999	30 (13.8)
	40,000-49,999	28 (12.9)
	50,000-59,999	29 (13.4)
	60,000-69,999	22 (10.1)
	70,000-79,999	14 (6.5)
	80,000-89,999	14 (6.5)
	90,000-99,999	9 (4.2)
	100,000 or more	21 (9.7)

**Table 2:** Summary of participant reponses to theory questions.

Question^a^	Response (N=217), n (%)				
	Strongly disagree	Disagree	Neutral	Agree	Strongly agree
Increased my belief that poor diet/nutrition leads to disease^b,c^	4 (1.8)	35 (16.1)	45 (20.7)	94 (43.3)	39 (18.0)
Increased my belief that eating a healthy diet can prevent disease^b,c^	4 (1.8)	22 (10.1)	34 (15.7)	93 (42.9)	64 (29.5)
Increased my belief that diseases related to poor diet/nutrition are harmful^b,c^	4 (1.8)	22 (10.1)	40 (18.4)	76 (35.0)	75 (34.6)
Increase my belief that eating a healthy diet is important in preventing disease^b,c^	4 (1.8)	14 (6.5)	31 (14.3)	91 (41.9)	77 (35.5)
Increased my motivation to eat a healthy diet^b^	1 (0.5)	6 (2.8)	0 (0.0)	80 (36.8)	130 (59.9)
Increased my ability to eat a healthy diet^b^	4 (1.8)	8 (3.7)	18 (8.3)	92 (42.4)	95 (43.8)
Increased my confidence that I can eat a healthy diet^b^	1 (.5)	6 (2.8)	11 (5.0)	105 (48.4)	94 (43.3)
Increased my desire to eat a healthy diet^c^	0 (0.0)	2 (0.9)	12 (5.6)	94 (43.3)	109 (50.2)
Increased my intentions to eat a healthy diet^c^	1 (0.5)	1 (0.5)	5 (2.3)	88 (40.5)	122 (56.2)
Increased my attitudes about the importance of eating a healthy diet in preventing disease^c^	3 (1.4)	15 (6.9)	21 (9.7)	97 (44.7)	81 (37.3)
Increased my belief that people important to me want me to eat a healthy diet^c^	9 (4.2)	33 (15.2)	52 (24.0)	66 (30.4)	57 (26.2)
Increased my perception that many other people are eating a healthy diet^b^	5 (2.3)	41 (18.9)	46 (21.2)	69 (31.8)	56 (25.8)
Increased my knowledge of the diseases that are caused by poor diet/ nutrition^b^	14 (6.5)	44 (20.3)	42 (19.3)	73 (33.6)	44 (20.3)
Increased my knowledge of the ways in which I can eat a healthy diet^b^	1 (.5)	7 (3.2)	14 (6.4)	98 (45.2)	97 (44.7)
Increased my awareness of the benefits of eating a healthy diet^d^	1 (0.5)	12 (5.5)	30 (13.8)	95 (43.8)	79 (36.4)
Increased my desire to be healthy^c^	1 (0.5)	5 (2.3)	8 (3.7)	78 (35.9)	125 (57.6)
Increased the social support I have received for eating a healthy diet^b^	11 (5.0)	41 (18.9)	39 (18.0)	78 (35.9)	48 (22.1)
Increased the positive feedback I have received for eating a healthy diet^b^	12 (5.5)	20 (9.2)	41 (18.9)	89 (41.0)	55 (25.4)
Increased my desire to set goals to eat a healthy diet^c^	0 (0.0)	0 (0.0)	5 (2.3)	84 (38.7)	128 (59.0)
Increased my ability to achieve my healthy diet goals^b^	0 (0.0)	1 (0.5)	11 (5.1)	94 (43.3)	111 (51.1)

^a^All theory questions in the survey were preceded by this statement: Now think about the diet/nutrition app(s) that you have used in the past 6 months. Using the app(s) has…

^b^Questions were derived from the social cognitive theory.

^c^Questions were derived from the theory of planned behavior.

^d^Questions were derived from the health belief model.

The majority of participants strongly agreed that using diet/nutrition apps led to changes in their behavior, namely increases in actual goal setting to eat a healthy diet (58.5%, 127/217), increases in their frequency of eating healthy foods (57.6%, 125/217), and increases in their consistency of eating healthy foods (54.4%, 118/217; Table 3).

Participants strongly agreed that the diet/nutrition apps they used were easy to use (62.9%, 156/217) and helpful (60.5%, 150/217; Table 4). Many also strongly agreed that they liked the apps (54.4%, 135/217) and enjoyed using the apps (44.8%, 111/217). About half of the participants (54.0%, 134/217) strongly agreed that they would recommend the apps to others. This scale, measuring engagement, received a high Cronbach alpha (.875).

Two of the predictors were significantly associated with theory (Table 5). Specifically, app engagement (*P*<.001) and app price (*P*<.01) were associated with theory.

Several predictors were also positively associated with diet-related behavior change (Table 6). Theory (*P*<.001), app engagement (*P*<.001), app use (*P*<.003), and education (*P*<.01) were all positively associated with behavior change.

**Figure 1:**
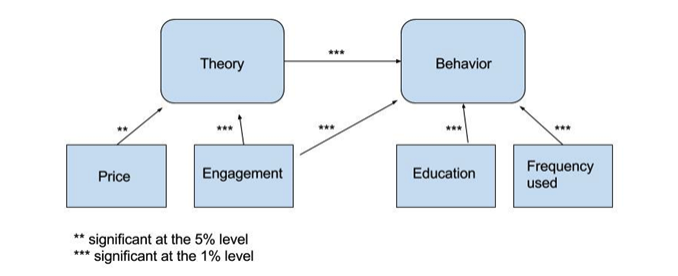
Factors influencing behavior change. Figure 1 illustrates the relationship between mobile application attributes, theoreticaldeterminants of behavior and behavior. Arrows indicate the hypothetical direction of therelationships and stars indicate the statistical significance.

Figure 1 summarizes significant relationships between theory, behavior, and other predictors. Arrows indicate the hypothetical direction of the relationships and asterisks indicate the level of statistical significance. In the model, the price of the app and the level of participant engagement affect theory, which in turn drives behavior change. Engagement also affects behavior directly. Finally, the level of participant education and the frequency of app use have a direct impact on behavior change.

**Table 3:** Summary of participant responses to behavior change questions.

Question^a^	Response (N=217), n (%)				
	Strongly disagree	Disagree	Neutral	Agree	Strongly agree
Increased my actual goal setting to eat a healthy diet	1 (0.5)	0 (0.0)	11 (5.1)	78 (35.9)	127 (58.5)
Increased my frequency of eating healthy foods	0 (0.0)	2 (0.9)	8 (3.7)	82 (37.8)	125 (57.6)
Increased my consistency in eating healthy foods	0 (0.0)	1 (0.5)	8 (3.7)	90 (41.4)	118 (54.4)

^a^All theory questions in the survey were preceded by this statement: Now think about the diet/nutrition app(s) that you have used in the past 6 months. Using the app(s) has…

**Table 4:** Summary of participant responses to engagement questions.

Question^a^	Response (N=217), n (%)				
	Strongly disagree	Disagree	Neutral	Agree	Strongly agree
The app(s) was helpful	2 (0.8)	3 (1.2)	4 (1.6)	89 (35.9)	150 (60.5)
The app(s) was easy to use	1 (0.4)	2 (0.8)	6 (2.4)	83 (33.5)	156 (62.9)
I enjoyed using the app(s)	2 (0.8)	4 (1.6)	26 (10.5)	105 (42.3)	111 (44.8)
I liked the app(s)	1 (0.4)	3 (1.2)	10 (4.0)	99 (39.9)	135 (54.4)
I would recommend the app(s) to others	1 (0.4)	3 (1.2)	19 (7.7)	91 (36.7)	134 (54.0)

^a^All engagement questions in the survey were preceded by this statement: Considering the diet/nutrition app(s) that you have used in the past 6 months…

**Table 5:** Ordinary least squares regression results for determinants of theory.

Determinants of theory	Coefficient (Standard error)	*t*	*P*>| *t* |	95% CI
App engagement	1.06 (0.17)	6.42	<.001	0.74-1.39
Price of app	0.50 (0.20)	2.50	.01	0.11-0.90
Frequency of app use	0.11 (0.17)	0.64	.52	−0.23 to 0.45
Gender	0.16 (0.17)	0.98	.33	−0.17 to 0.50
Age	0.05 (0.11)	0.49	.63	−0.16 to 0.26
Income	−	−0.66	.51	−0.17 to 0.09
Education	−	−1.36	.18	−0.11 to 0.02
Constant	1.33 (0.61)	2.20	.03	0.14-2.53

**Table 6:** Ordinary least squares regression results for determinants of behavior change.

Independent Variables	Coefficient (Standard error)	*t*	*P*>| *t* |	95% CI
Theory	0.10 (0.02)	4.87	<.001	0.06-0.14
App engagement	0.39 (0.05)	7.56	<.001	0.29-0.50
Price of app	−0.06 (0.06)	−1.02	.31	−0.18 to 0.06
Frequency of app use	0.15 (0.05)	3.02	.003	0.05-0.25
Gender	0.05 (0.05)	0.96	.34	−0.49 to 0.14
Age	−0.00 (0.03)	−0.14	.89	−0.06 to 0.06
Income	0.01 (0.01)	0.88	.38	−0.01 to 0.03
Education	0.05 (0.02)	2.60	.01	0.01-0.09
Constant	0.20 (0.18)	1.14	.26	−0.15 to 0.55

## Discussion

### Principal Findings

The purpose of this study was to explore behavior change mechanisms associated with the use of diet- and nutrition-related health apps and to examine if the use of these apps is associated with actual changes in dietary behaviors. The results of this study demonstrate that the use of diet/nutrition apps is associated with diet-related behavior change. In addition, this study showed that behavior change theory was positively associated with actual behavior change related to the use of diet/nutrition apps.

Participants in this study reported increased motivation, desire, and ability to improve their dietary intake with app use. Likewise, participants indicated an increase in their ability to establish and achieve dietary goals. Taken together such increases indicate that diet- and nutrition-related apps improve self-efficacy, or strengthen one’s belief that they can engage in healthy dietary behaviors. Self-efficacy is a key component of SCT and is widely considered to be a powerful predictor of health behavior and appears to be a key mechanism by which health apps facilitate behavior change [8,22,23].

Survey results also indicate that app use helped to create attitudes supportive of improved dietary behaviors, as well as behavioral intentions to eat a healthy diet. The TPB postulates that behavioral attitudes and beliefs coupled with subjective norms and self-efficacy predict behavioral intentions [13]. Results from this study indicate that attitudes related to the importance of eating healthy and the subsequent behavioral intentions of doing so are mechanisms for behavior change when using diet- and nutrition-related apps. This dovetail between self-efficacy and enhanced autonomy has been observed in other studies where behavior change was achieved through the use of mobile health informatics tools that are patient-centered and increase self-management skills [11,24].

Finally, participants reported an increase in knowledge of the ways in which they can eat a healthy diet and an awareness of the benefits of improving dietary habits. Though general knowledge alone is often an insufficient change agent [25], this study demonstrates that knowledge specifically related to ways in which one can improve dietary behavior and the benefits of making such improvements are mechanisms for change when using diet- and nutrition-related apps.

### Limitations

Several limitations should be considered when interpreting the findings of this study. First, this study has limited racial and ethnic diversity among participants. Respondents in this study were primarily white with similar levels of age, education, and economic status. This limitation is likely a reflection of the demographic using MTurk’s Web-based surveying system [26]. Additional research is needed with a more diverse sample to make generalizations related to this study’s key findings.

Second, this study included only limited data on participants due to the need to balance resource constraints with various research questions of interests. The study would be strengthened by collecting additional participant data and information. For example, collecting information regarding respondents’ height and weight to examine relationships between health outcomes such as body mass index and diabetes and app use may have been helpful. Third, a pre- and posttest design evaluating participants’ dietary and nutritional behaviors before and after downloading the app may reveal additional insight into mechanisms for behavior change related to app use. Future studies may benefit from qualitative research designs targeting motivations of diet/nutrition app use over time. Fourth, understanding participants’ motivations for downloading and using diet- and nutrition-related apps may also have been useful, as would have determining whether or not those apps met participants’ expectations. Despite these limitations, this study represents an initial effort to understand the mechanisms by which diet- and nutrition-related apps lead to behavior change, which can guide both future app development and research design.

### Conclusions

Diet- and nutrition-related mobile apps show promise as tools to successfully facilitate positive health behavior change. The results of this study confirm that the use of diet/nutrition apps is associated with diet-related behavior change. Furthermore, apps that focus on improving motivation, desire, self-efficacy, attitudes, knowledge, and goal setting may be particularly useful. To ensure that mobile apps are effective health behavior change agents, theories and their respective constructs known to facilitate health behavior change, such as those of SCT, TPB, and HBM, should continue to be integrated into health app design and implementation. Moving forward, developers of diet/nutrition apps may consider design configurations that emphasize the provision of knowledge to shape attitudes and beliefs, followed by attempts to influence actual skill development in app users. Elements of gamification or other such paradigms may be useful to maintain user motivation and the desire to be persistent in making weight loss efforts.

## Abbreviations

HBM: health belief model
SCT: social cognitive theory
TPB: theory of planned behavior
